# A Brief Analysis of Traditional Chinese Medical Elongated Needle Therapy on Acute Spinal Cord Injury and Its Mechanism

**DOI:** 10.1155/2013/828754

**Published:** 2013-11-24

**Authors:** Mengxuan Du, Rongliang Chen, Renfu Quan, Liang Zhang, Jinwei Xu, Zhongbao Yang, Disheng Yang

**Affiliations:** ^1^Research Institute of Acupuncture and Moxibustion, Xiaoshan Traditional Chinese Medical Hospital, Zhejiang 311200, China; ^2^Research Institute of Acupuncture and Moxibustion, Zhejiang Chinese Medical University, Hangzhou 31012, China; ^3^Research Institute of Acupuncture and Moxibustion, Medical College of Xiamen University, Xiamen 361005, China; ^4^Department of Acupuncture and Moxibustion, The Second Affiliated Hospital, Medical College of Zhejiang University, Hangzhou 310009, China

## Abstract

Acute spinal cord injury is one of the most common and complicated diseases among human spinal injury. We aimed to explore the effect of point-through-point acupuncture therapy with elongated needles on acute spinal cord injury in rabbits and its possible mechanism. Adult rabbits were randomly divided into a model group, elongated needle therapy group, and blank group. Immunohistochemical staining showed that the protein levels of Fas and caspase-3 in the model group were significantly higher than those in the blank group at each time point (*P* < 0.05) and significantly lower than those in the elongated needle therapy group on the 3rd and 5th days after operation (*P* < 0.05). RT-PCR showed that Fas and caspase-3 mRNA levels in the model group and elongated needle therapy group were significantly higher than those in the blank group (*P* < 0.05, 0.01). The mRNA levels of Fas and caspase-3 in the elongated needle therapy group were significantly lower than those in model group on the 3rd day (*P* < 0.05, 0.01). Therefore, we confirmed that elongated needle therapy has an obvious effect on acute spinal cord injury in rabbits. Its mechanism is made possible by inhibiting the expression of the Fas→caspase-3 cascade, thereby inhibiting cell apoptosis after spinal cord injury.

## 1. Introduction

Traditional Chinese medicine has centralized Chinese medical wisdom from all ages, which has led to a huge quantity of information. Acupuncture is a traditional Chinese medical therapy that uses elongated needles and modern penetration needling. This therapy has excellent results in both clinical care and acupuncture studies. Compared with other medical skills in China and abroad, elongated needle therapy is famous for its better economic efficiency, progressiveness, and representativeness.

The symptoms, morbidity, conditions, pathogenesis, and pathology of spinal cord injury (SCI) are complex. SCI is one of the most serious diseases endangering human health and its mechanisms are complicated. Recent studies have found that apoptosis and various genes have a role after spinal cord injury [1]. There are clinical reports [2, 3]. That claim adopting acupuncture to treat spinal cord injury has a curative effect. However, its curative mechanism is still unclear. We used a modified Allen's film forming method of spinal cord injury to build a spinal cord jury model called rabbit T13-L1 and coupled it with elongated needling acupuncture point therapy. Then through shape changes of the rabbit spinal cord after injury, we tested the Fas receptor for apoptosis, protein Caspase-3, and the expression of mRNA.

## 2. Materials and Methods

### 2.1. Model Establishment

Urethane at 20%, 1 g/kg was injected into the ear vein for anesthetization. Then, rabbits were fixed in the prone position on the operating table, and under sterile conditions, with T13 as the center, the middle of rabbit's back was cut open about 2-3 cm. The T13-L1 spinous process and all the vertebral plates were broken off, and a 3 mm wide dura mater was exposed. A modified Allen's striking device was used to impact the T13-L1 spinal cord with an 80 gram cm force. Successful model establishment was considered when there was spastic swinging of the tail, fluttering of both lower extremities and body, and lower extremity flaccid paralysis. After operation, Crede's manipulation was used to massage the rabbit's abdomen every day to help defecation and uresis 2–4 times. For three consecutive days, 800,000 units of penicillin were injected to prevent wound infection.

### 2.2. Animal Preparation and Group of Experiments

Eighty-four purebred Japanese rabbits, of unknown gender, body weight 2.5 ± 0.25 kg were obtained from the Zhejiang Chinese Medical University Animal Experiment Research Center. Rabbits were randomly assigned into three groups: a model group, in which the SCI model was established without elongated needle therapy; elongated needle group; and control group. For elongated needle therapy, both sides of elongated needles were used to penetrate “Chihpien,” “Flume,” “Qihai,” and “Intermediate” daily. Meanwhile, “Chihpien” and “Flume” formed a loop and were connected with a JL2B electric pulse stimulator for 15 min at a rate of 20–40 beats/min at an intensity of 1.5–3 V. For the sham operation group, we operated on the T13-L1 spinous process and all vertebral plates, and a 3 mm wide dura mater was opened but the spinal cord was not touched and there was no electrical stimulation. We found the acupuncture point locations for experimental animals in the “*Subject of Experimental*.”

### 2.3. Slice Preparation

Each animal group was sacrificed on the 1st, 3rd, and 5th days after operation. After anesthesia, a longitudinal cut was made in the middle of chest; the heart was exposed. Then, the left ventricle and right atrium were opened, and from the left ventricle, a tube was inserted along the aorta. Then, physiological saline was injected until the perfusate became clear and bright. Next, the heat was rapidly filled with 4% paraformaldehyde PD solution (pH = 7.4) at a flow rate of 50 mL/min for 3-4 min, followed by a flow rate of 10 mL/min for 20 min. Later, a 1 cm T13-L1 vertebral segment of spinal cord was removed. Finally, 4% paraformaldehyde solution was used to fix specimens for 24–48 h. Samples were routinely embedded in paraffin and sliced at a thickness of 4 *μ*m for preservation.

### 2.4. Hematoxylin and Eosin (HE) Dyeing

Slices were taken from each rabbit randomly and stained normally with hematoxylin and eosin. Then, the shape changes of the spinal cord were observed through a light microscope.

### 2.5. Immunohistochemical Staining Method (SP)

Sections from each rabbit were randomly selected and dyed according to the manufacturer's instruction. The antibody concentration of Fas was 1 : 100 and caspase-3 was 1 : 4000. DAB coloration was used with hematoxylin as a counterstain. Xylene was used to make tissue transparent and sections were mounted. PBS was used as a negative control. Both the positive cells and endochylema are pale brown. With an OLYMPUS BX-50 light microscope at 400 fields, three fields were randomly chosen per section to observe the expression of all positive cells in the anterior and posterior horns of spinal cord (there are cells of pale brown in endochylema and karyon). Image analysis software Image-Pro Plus was then used to count the optical density.

### 2.6. Semiquantitative RT-PCR to Testify the Expression of Fas and Caspase-3 mRNA

Three rabbits were chosen from each group at each time point and TRIzol (Invitrogen, Carlsbad, CA, USA) was used to extract total RNA of each spinal cord tissue. OD was also determined. A reverse transcription kit (all from Applied Biosystems, Foster City, CA) was used to make cDNA, and PCR amplification was performed. Gre-pro was used to analyze the electrophoresis grey level of Fas and caspase-3 mRNA and calculate relative expression. Relative expression amount = experimental grey level/internal reference grey level.

According to GenBank sequences, we used Primer 5.0 software (Premier company, Canada) to design primers for Fas and caspase-3. Primer sequences are shown in Table 1. *β*-actin was used as an internal reference.

### 2.7. Statistical Treatment

Statistical software SPSS17.0 (SPSS company, Chicago, IL, USA) was used to analyze data, and results are presented as $\overset{-}{x}\pm s$. Variance analysis and LSD were used. When *P* < 0.05, the difference was considered statistically significant.

## 3. Experimental Results

### 3.1. Result of HE Staining

**Figure 1:**
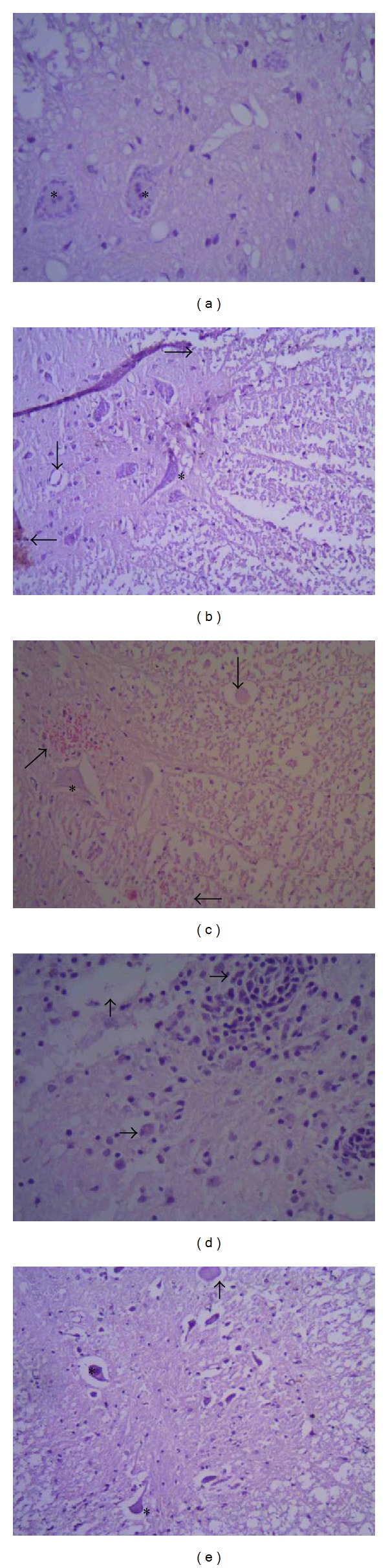
(a) On the 5th day after sham operation, a gray-white boundary of the spinal cord and the nuclei of the neurons were shaped like waves with two sharp peaks; glial cells were scattered; and nuclear Nissl body were clear (HE ×400). (b) On the 1st day after model group operation, the normal structure of the spinal cord tissue was lost; there was hemorrhaging, edema, and necrosis; the boundary between the grey and white matter was unclear; part of the neuronal nuclei decreased; and there was cavity disappearance. Glial cells proliferated, and there was local lymphocytic infiltration (HE ×200). (c) On the 1st day after elongated needle therapy, the normal structure of the spinal cord tissue was lost; there was hemorrhaging, edema, necrosis, and demyelination; the boundary between the grey and white matter was unclear; part of the neuronal nuclei decreased, and gliocytes proliferated around damaged areas (HE ×200). (d) On the 5th day after model group operation, the structure of the spinal cord tissue was disorganized; there was serious karyopyknosis; and a bigger cavity was formed with excessive gliocyte proliferation (HE ×400). (e) On the 5th day after elongated needle therapy, there were still some complete neuronal nuclei. There was a clear boundary between grey and white matter and less gliocyte proliferation (HE ×200).

On the 5th day after sham-operation, a gray-white boundary of the spinal cord and the nuclei of the neurons were shaped like waves with two sharp peaks, glial cells were scattered, and nuclear Nissl body were clear (HE ×400, Figure 1(a)). On the 1st day after model group operation, the normal structure of the spinal cord tissue was lost; there was hemorrhaging, edema, and necrosis; the boundary between the grey and white matter was unclear; part of the neuronal nuclei decreased; and there was cavity disappearance. Glial cells proliferated, and there was local lymphocytic infiltration (HE ×200, Figure 1(b)). On the 1st day after elongated needle therapy, the normal structure of the spinal cord tissue was lost; there was hemorrhaging, edema, necrosis, and demyelination; the boundary between the grey and white matter was unclear; part of the neuronal nuclei decreased; and gliocytes proliferated around damaged areas (HE ×200, Figure 1(c)). On the 5th day after model group operation, the structure of the spinal cord tissue was disorganized, there was serious karyopyknosis, and a bigger cavity was formed with excessive gliocyte proliferation (HE ×400 Figure 1(d)). On the 5th day after elongated needle therapy, there were still some complete neuronal nuclei. There was a clear boundary between grey and white matter, less gliocyte proliferation (HE ×200, Figure 1(e)), and no bleeding.

### 3.2. Results of Immunohistochemistry

The results of Fas and caspase-3 optical density values of rabbit spinal cord tissue among each group at different time points is presented in Table 2. Positive expression of each sample point in the model group was significantly different than the sham operation group. Positive expression of each Fas albumen sampling point in elongated needle group was significantly different than the model group. Positive expression of caspase-3 on the 3rd and 5th days after operation in the elongated needle group was significantly different than with the model group.

#### 3.2.1. Results of Fas Immunohistochemistry

**Figure 2:**
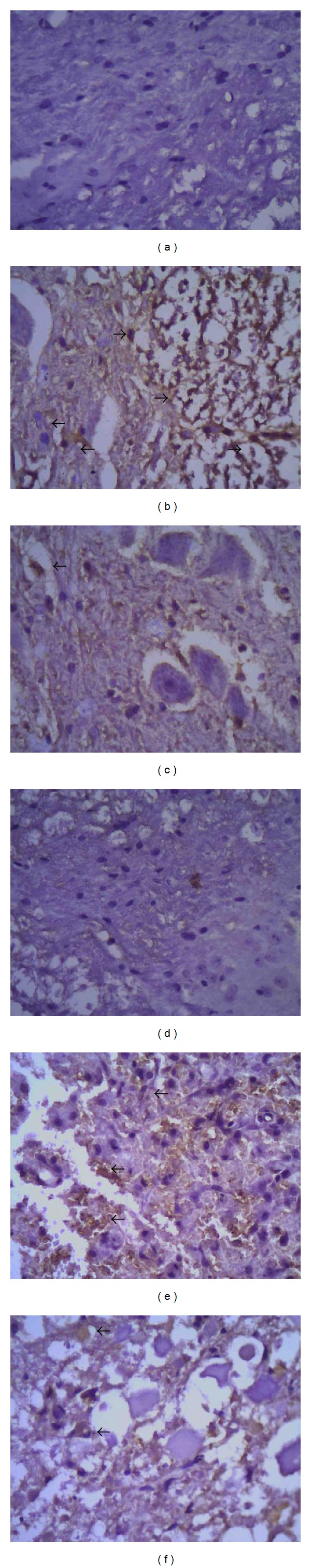
Use immunohistochemistry staining method to observe the positive cells expression in anterior and posterior horns of spinal cord. (a) On the 3rd day in the sham operation group, there was little positive cells expression of Fas protein (SP ×400). (b) On the 3rd day in the model operation group, positive cells of Fas protein scatter on damaged grey and white neuron, most of the positive cells scatter on white matter. There are positive signals inside ectoenzyme and endochylema (SP ×400). (c) On the 3rd day in the elongated needle therapy, the grey matter of Fas protein positive cells are less when comparing with the model group (SP ×400). (d) On the 5th day in the sham operation group, there is no evidence to show the positive cells expression of Caspase-3 protein. (e) On the 5th day in the model operation group, Caspase-3 protein positive cells are scattered on damaged grey matter dorsal horn neurons, and there are positive signals inside ectoenzyme and endochylema (SP ×400). (f) On the 5th day in the elongated needle therapy, there is expression of Caspase-3 positive cells in dorsal horn region of spinal cord grey matter, but the expression of positive cells is more scattered than that of the model group (SP ×400).

There was little positive expression of each sampling point in the sham operation group (SP ×400; Figure 2(a)). Some positive expression appeared on the 1st day after operation in both the model and elongated needle groups (Table 2). Expression reached a peak on the 3rd day after operation. The positive cells in the model group were scattered with damaged grey and white neurons, and the inner white matter expressed more positive cells. The positive signal was found inside ectoenzymes and endochylema (SP ×400; Figure 2(b)). The elongated needle group had a much smaller expression area of visible positive cells in the grey matter than that in the model group (SP ×400, Figure 2(c)).

#### 3.2.2. Results of Caspase-3 Immunohistochemistry

There was little positive expression of each sampling point in the sham-operation group (SP ×400; Figure 2(d)). There was positive expression on the 1st day after operation in both the model and elongated needle groups (Table 2). Expression reached a peak on the 3rd day after operation. The positive cells in the model group were mainly scattered in the damaged dorsal horn grey matter. The positive signal was found inside ectoenzymes and endochylema (SP ×400; Figure 2(e)). In the elongated needle group, there was positive expression in the dorsal horn region of the spinal cord grey matter. However, the expression of positive cells was more scattered than that of the model group (SP ×400; Figure 2(f)).

#### 3.2.3. Results of Semiquantitative RT-PCR for Expression of Fas and Caspase-3 mRNA

**Figure 3:**
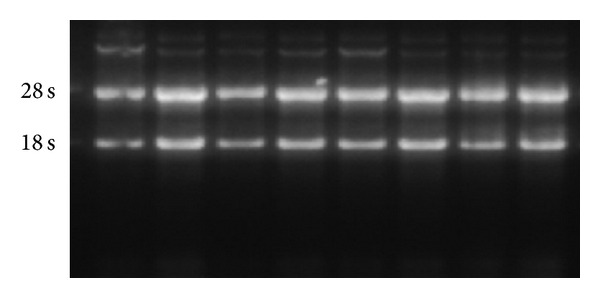
RNA extraction integrity.

**Figure 4:**
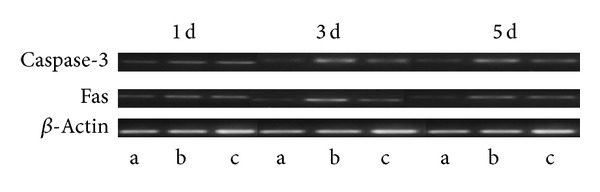
Fas mRNA and Caspase-3mRNA electrophoresis. *Note*: a: sham-operation group, b: model group, and c: elongated needle group.

Table 3 shows the expression of Fas and caspase-3 mRNA after spinal cord injury. The integrity of RNA extraction is shown in Figure 3. The results of the electrophoresis are presented in Figure 4. Fas and caspase-3 mRNA expressions of each sampling point in the model and elongated needle groups significantly increase more than in the sham operation group. The expression of Fas mRNA on the 1st and 3rd days after operation in the elongated needle group is significantly different than that in the model group. The expression of caspase-3 mRNA on the 3rd and 5th days after operation in the elongated needle group significantly decrease more than in the model group.

## 4. Discussion and Analysis

 The term “elongated needle” is derived from the long needle of the “nine classical needles” in *Huangdi Neijing* (the internal canon of medicine), famous for its wheat-like shape. Elongated needles are the thinnest needle in acupuncture, and penetration needling is a novel style of deep needling. Elongated needle therapy could help dredge Qi-blood of internal organs and main and collateral channels. Then, through the induction of main and collateral channels, Qi reaches the affected area. The effect of this therapy reaches beyond ordinary filiform needles. Mao [4] has summarized clinical reports of using elongated needle therapy and states that the emphasis of elongated needle therapy should be on deep penetration. Thus, elongated needle therapy applies to diseases of deep nerves, muscles and ligaments. The hand-manipulating route of Chihpien and Flume, accompanied with vascular anatomy, could confirm that ample blood vessels and nerve tracts that exist in the pelvic autonomic nerve. Elongated needles used to stimulate the pelvic autonomic nerve can adjust bladder detrusor and urethra sphincterismus, and it also has an effect on coordinating muscles. Moreover, it can reach the spinal center or cerebral cortex, and this conduction of neural signal has an influence on repairing damaged spinal cord tissue.

In our experiment, we found that nerve cell apoptosis co exists with necrosis after spinal cord injury. During the primary period of damage, there was organelle damage or dissolving, regional structure in a mass, and apoptosis, karyopyknosis, and apoptotic body formation. There were also one or several apoptotic areas around the spinal cord tissue. Therefore, apoptosis is one of the factors causing lesion area expansion, which agrees with previous studies [5, 6]. Using elongated needles to penetrate “Chihpien-Flume” points could save more spinal cord injury tissue neurons than that of the model group. The technique also clarifies nerve cell structure and decreases edema and necrosis, compared with the model group. There is also less proliferation of glial cells, less Fas and caspase-3 protein expressions, and less Fas and caspase-3 mRNA expressions in the elongated needle group than in the model group. Therefore, the elongated needle technique is effective at curing spinal cord injury, and apoptosis reduction is one of mechanisms of its curative effect.

Spinal cord injury includes two types, one is primary spinal cord injury and the other is sequential spinal cord injury. Primary spinal cord injury is caused by mechanical pressure, hemorrhage, or electrolytic changes inside cells. Primary spinal cord injury occurs in several hours and is irreversible. Sequential spinal cord injury refers to a series of biochemical changes, which include ischemia reperfusion, inflammation, apoptosis, immune response, blood spinal cord barrier damage, intracellular and extracellular ion disturbance, and free radical reaction. The delayed death of nerve cells from spinal cord injury may be reversible [7]. Medications, physicotherapeutics, and operations can treat secondary damage after SCI and can promote the regeneration of the spinal cord. The effective treatment of sequential spinal cord injury can improve patient prognosis and quality of life [8]. Therefore, nerve recovery after sequential spinal cord injury is also the focus of our research.

Apoptosis is an essential part of sequential spinal cord injury. From the pathological point of view, mechanical force can cause bleeding, which is followed by tissue angiospasm, thrombus formation inside capillaries and ischemia in local nerve tissue. Mechanical force and ischemia in local nerve tissues can reinjure capillary endothelial cells, which causes expression of inflammatory mediators and activation of the glial cells and monocyte macrophage system. Then, inflammatory cells aggregate and clog capillaries, which aggravates the local ischemia [9]. After spinal cord injury, changes in the stability of factors that participate in pathological changes, such as oxygen free radicals and excitatory amino acids, could arouse oxygen free radical reaction, lipid peroxidation, and excitotoxicity. This can lead to changes in cell membrane permeability, intracellular calcium, sodium ion overload, and cellular edema [10]. These changes can eventually lead to apoptosis. Massive expression of inflammatory mediators and tumor necrosis factors can also induce apoptosis in the death receptor Fas/TNFR mediated pathway [11].

Fas is a 45 kDa membrane protein receptor, which belongs to tumor necrosis factor receptor family (TNFR) [12]. Fas can combine with caspase-8 to form a death-inducing signaling complex. Therefore, the expression of Fas can promote apoptosis [13]. The common pathway in the anaphase of apoptosis is caspase activation. Cysteine proteinase caspase functions as a final hub in apoptosis with several activated factors. Activated caspase-3 can cut many protein substrates, including cytoskeletal proteins (*α*-spectrin, *β*-spectrin, actin, and tau proteins), proteins participating in the regulation of the cytoskeleton, and enzymes participating in DNA repair such as PARP, DNA-PKcs, and apoptosis albumen Bcl-2. This finally may lead to cell death [14, 15]. In our experiments, we observed less expression of Fas and caspase-3 protein and weaker Fas and caspase-3 mRNA expressions in semiquantitative RT-PCR than that of the model group. Fas mRNA expression on 1st, 3rd, and 5th days in the model group are 2.1, 4.0 and 3.4 times that of the sham operation group. Through statistical analysis, this corresponds to homogeneity of variance. We observed that the peak-expression of Fas mRNA is earlier than that of caspase-3 mRNA. This shows that the mechanism of elongated needle therapy is to firstly activate Fas, and after a cascade of reactions, activate the caspase-3 cascade. Elongated needle acupuncture has a curative effect on spinal cord injury, and restraining apoptosis is one of its mechanisms. The Fas→caspase-3 cascade is probably the method by which apoptosis is restrained after spinal cord injury when using elongated needles. This provides new evidence for SCI and provides new clues to curing spinal cord injury.

## 5. Conclusions

Encouraging comprehensive treatment is the common clinical method for treatment of SCI, and acupuncture as a traditional and economic skill has an effect in curing spinal cord injury [16]. This study attempts to build up a new theoretical base of using elongated needle therapy to cure spinal cord injury.

## Figures and Tables

**Table 1:** Gene primer sequence table of Fas and Caspase-3.

Gen	Primer	Oligonucleotide sequences (5′-3′)	Length (bp)
Fas	Front	5′-GCAGACAAGCGATTTACTTCT-3′	581
	Rear	5′-ATCAGAACAGTGAAGCGTACA-3′	
Caspase-3	Front	5′-GCTGTGTGGGCTTGCTAAGTT-3′	749
	Rear	5′-TCACGTATCCTGGCGACTGTC-3′	
*β*-actin	Front	5′-GAGTACGCCAACATGGTGCTGTC-3′	146
	Rear	5′-CGTTCATGAGCCACACTTAGC-3′	

**Table 2:** The Fas and Caspase-3 optical density values of rabbit spinal cord tissue among each group at different time points.

Group	*n*	Fas			Caspase-3		
		1 d	3 d	5 d	1 d	3 d	5 d
Sham-operation group	9	0.040 ± 0.009	0.041 ± 0.010	0.049 ± 0.009	0.0412 ± 0.0217	0.0247 ± 0.0129	0.0358 ± 0.0125
Model group	9	0.372 ± 0.047^••^	0.743 ± 0.068^••^	0.256 ± 0.040^•^	0.4050 ± 0.0554^••^	0.7468 ± 0.0486^••^	0.2723 ± 0.0404^••^
Elongated needle group	9	0.314 ± 0.089^▲^	0.465 ± 0.045^▲▲^	0.216 ± 0.063^▲^	0.3935 ± 0.0767	0.5540 ± 0.0439^▲▲^	0.226 ± 0.0749^▲^

compared with sham-operation group ^•^ *P* < 0.05 and ^••^ *P* < 0.001; compared with model group ^▲^ *P* < 0.05 and ^▲▲^ *P* < 0.01.

**Table 3:** The FasmRNA and Caspase-3mRNA relative expression level of rabbit spinal cord tissue among each group at different time points.

Group	*n*	Fas			Caspase-3		
		1 d	3 d	5 d	1 d	3 d	5 d
Sham-operation group	9	0.1034 ± 0.0085	0.1125 ± 0.0133	0.1357 ± 0.0272	0.1906 ± 0.0290	0.2176 ± 0.0132	0.1928 ± 0.0150
Model group	9	0.5069 ± 0.0687^••^	0.7223 ± 0.0257^••^	0.3147 ± 0.0142^•^	0.4074 ± 0.0310^••^	0.8583 ± 0.0402^••^	0.6534 ± 0.0273^••^
Elongated needle group	9	0.4075 ± 0.0216^▲^	0.5992 ± 0.0396^▲^	0.2834 ± 0.0216	0.4289 ± 0.0151	0.6236 ± 0.0223^▲▲^	0.3478 ± 0.0258^▲▲^

Note: compared with sham-operation group ^•^ *P* < 0.05, ^••^ *P* < 0.001; compared with model group ^▲^ *P* < 0.05, ^▲▲^ *P* < 0.01.
